# Reduced surface pH and upregulated AE2 anion exchange in SLC26A3-deleted polarized intestinal epithelial cells

**DOI:** 10.1152/ajpcell.00590.2023

**Published:** 2024-01-15

**Authors:** Mahdi Amiri, Min Jiang, Azam Salari, Renjie Xiu, Seth L. Alper, Ursula E. Seidler

**Affiliations:** ^1^Department of Gastroenterology, Hannover Medical School, Hannover, Germany; ^2^Division of Nephrology, Beth Israel Deaconess Medical Center, Boston, Massachusetts, United States; ^3^Department of Medicine, Harvard Medical School, Boston, Massachusetts, United States

**Keywords:** alkalosis, bicarbonate secretion, Caco2BBe, chloride-losing diarrhea, intestine, pHi-regulation

## Abstract

Loss of function mutations in the *SLC26A3* gene cause chloride-losing diarrhea in mice and humans. Although systemic adaptive changes have been documented in these patients and in the corresponding knockout mice, how colonic enterocytes adapt to loss of this highly expressed and highly regulated luminal membrane anion exchanger remains unclear. To address this question, *SLC26A3* was deleted in the self-differentiating Caco2BBe colonic cell line by the CRISPR/Cas9 technique. We selected a clone with loss of SLC26A3 protein expression and morphological features indistinguishable from those of the native cell line. Neither growth curves nor development of transepithelial electrical resistance (TEER) differed between wild-type (WT) and SLC26A3 knockout (KO) cells. Real-time qPCR and Western analysis in SLC26A3-KO cells revealed an increase in AE2 expression without significant change in NHE3 expression or localization. Steady-state pH_i_ and apical and basolateral Cl^−^/HCO_3_^−^ exchange activities were assessed fluorometrically in a dual perfusion chamber with independent perfusion of luminal and serosal baths. Apical Cl^−^/HCO_3_^−^ exchange rates were strongly reduced in SLC26A3-KO cells, accompanied by a surface pH more acidic than that of WT cells. Steady-state pH_i_ was not significantly different from that of WT cells, but basolateral Cl^−^/HCO_3_^−^ exchange rates were higher in SLC26A3-KO than in WT cells. The data show that CRISPR/Cas9-mediated *SLC26A3* deletion strongly reduced apical Cl^−^/HCO_3_^−^ exchange rate and apical surface pH, but sustained a normal steady-state pH_i_ due to increased expression and function of basolateral AE2. The low apical surface pH resulted in functional inhibition of NHE-mediated fluid absorption despite normal expression of NHE3 polypeptide.

**NEW & NOTEWORTHY** SLC26A3 gene mutations cause chloride-losing diarrhea. To understand how colonic enterocytes adapt, SLC26A3 was deleted in Caco2BBe cells using CRISPR/Cas9. In comparison to the wild-type cells, SLC26A3 knockout cells showed similar growth and transepithelial resistance but substantially reduced apical Cl^−^/HCO_3_^−^ exchange rates, and an acidic surface pH. Steady-state intracellular pH was comparable between the WT and KO cells due to increased basolateral AE2 expression and function.

## Graphical abstract

**Figure:**
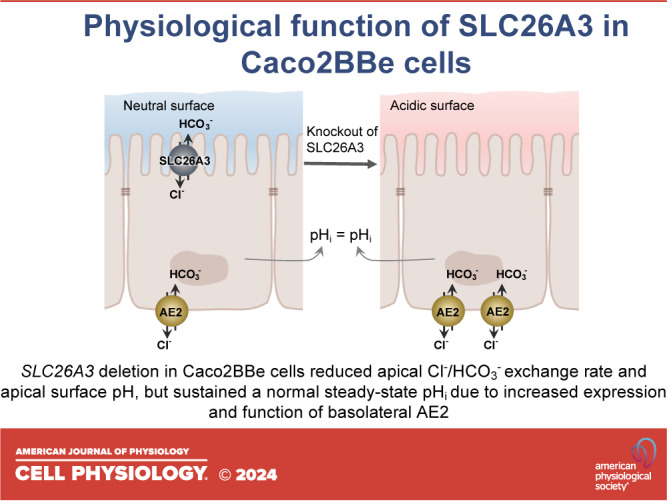

## INTRODUCTION

The Cl^−^/HCO_3_^−^ exchanger SLC26A3, also known as DRA, is strongly expressed in differentiated absorptive enterocytes of the mammalian intestine. The segmental expression pattern is species-dependent, but particularly high expression levels are present in the mid-distal colon in humans and in several rodent species (1–3). Loss of function mutations result in the disease “congenital chloride diarrhea”, or “chloride-losing diarrhea” (CLD), which is severely debilitating or lethal if not recognized and treated at birth (4, 5). *Slc26a3*^−/−^ mice suffer from most CLD features observed in humans, including the high susceptibility to intestinal inflammation (6, 7). Adaptive changes in CLD patients gradually reduce their dependence on intravenous supplementation of fluid and electrolytes (8).

Systemic hyponatremia, alkalosis and volume depletion in *slc26a3*^−/−^ mice stimulate hyperaldosteronism (6). The resulting profound hypokalemia may lead to sudden cardiac death. The hyperaldosteronism results in a massive colonic upregulation of the absorptive sodium transporter NHE3 (SLC9A3) and the epithelial sodium channel ENaC (6) However, whereas *slc9a3*^−/−^ mice maintain a pelleted stool despite hyperaldosteronism accompanied by colonic upregulation of ENaC, *slc26a3*^−/−^ mice and CLD patients have persistent, watery, chloride-rich stool leading (in mice) to premature death. The colonic mucosa of *slc26a3*^−/−^ mice harbors several additional abnormalities, including mucosal hyperplasia (6), a lack of firmly adherent mucus gel (7), paracellular barrier defects (9), a strongly dysbiotic fecal microbiome (10), a hyperactive protective response to intestinal pathogens by the host mucosa (11), and spontaneous development in adulthood of mild mucosal inflammation (10).

It is unclear, however, which of these abnormalities might be directly attributable to one or more of the base extruders of the differentiated colonocyte, and which might be secondary to compensatory activation of the renin-angiotensin-aldosterone system, to the dysbiotic microbiome, or to loss of the protective mucus layer (11, 12). We therefore deleted SLC26A3 in the self-differentiating Caco2BBe cell line by CRISPR/Cas9 and generated clonal cell lines lacking SLC26A3 protein. We selected for further study one SLC26A3-KO clonal cell line with transepithelial electrical resistance, ultrastructural morphology and tight junctional protein expression and localization resembling those of parental cells. This cell line was subjected to measurements of proliferative capacity, apical and basolateral anion exchange rates, extracellular surface pH and steady-state pH_i_ under equilibrium conditions.

## MATERIALS AND METHODS

### Cell Culture and Establishment of SLC26A3 Knockout and AE2 Knockdown Cell Clones

Caco2BBe1 cells (CRL-2102, ATCC) were cultured in Dulbecco’s modified Eagle’s medium (Gibco) supplemented with 10% FBS (Sigma-Aldrich), 1% nonessential amino acids (Gibco) and penicillin/streptomycin (Sigma-Aldrich) at 37°C with 5% CO_2_.

To establish SLC26A3 knockout cell lines, 5′- CACCGCATTCTTTAAGCCGGTATGC and 5′- AAACGCATACCGGCTTAAAGAATGC oligonucleotides (Sigma-Aldrich) were designed, annealed and cloned into pSpCas9(BB)-2A-GFP (Cat. No. 48138, Addgene, MA) according to Ran et al. (13). The final construct was verified by Sanger sequencing. Caco2BBe cells at 70–80% confluency were transfected with the CRISPR-Cas9 construct using lipofectamine 3000 reagent (Thermo Fisher Scientific), per manufacturer’s protocol. Two days posttransfection, the cells were detached by trypsin-EDTA solution (Sigma-Aldrich), washed and strained through a 70 µm cell strainer (Greiner Bio-One, Frickenhausen, Germany) and sorted using a FACSAria IIu Cell Sorter (BD Biosciences, Heidelberg, Germany). Single GFP-positive cells were collected in separate wells of a 96-well culture plate containing 100 µL complete culture medium supplemented with 20% FBS, previously conditioned for 24 h on postconfluent Caco2BBe monolayers, then passed through a 0.45-µm sterile filter to remove detached cells and stored for up to one week at 4°C. The conditioned medium was routinely exchanged in the 96-well plate until small colonies appeared. Thereafter, the colonies were maintained and expanded in normal complete medium and sampled to check for SLC26A3 knockout. Genomic DNA of the resulting clones as well that of wild-type Caco2BBe cells was isolated by DNA extraction kit (Qiagen), amplified by Phusion polymerase (Thermo Fisher Scientific) using the primers 5′- ACATCGCTGTCGCAAAACAC and 5′- GTATATCTGACAAGGGTCTGG, then purified using PCR clean up kit (Qiagen) and analyzed by Sanger sequencing (Microsynth Seqlab, Gottingen, Germany). Sequences were analyzed using Snapgene software version 4.2 (GSL Biotech, CA). Differentiated cultures of selected SLC26A3-KO clones were further compared with wild-type cells by Western blot.

Expression of AE2 (SLC4A2) protein in Caco2BBe cells was downregulated using the shRNA technique. For this purpose, 5′- CCGGAGACAGCTCGCTGGATCAAATCTCGAGATTTGATCCAGCGAGCTGTCTTTTTTG and 5′- AATTCAAAAAAGACAGCTCGCTGGATCAAATCTCGAGATTTGATCCAGCGAGCTGTCT oligonucleotides against *AE2* or the scrambled oligonucleotides 5′- CCGGCCTAAGGTTAAGTCGCCCTCGCTCGAGCGAGGGCGACTTAACCTTAGGTTTTTG and 5′- AATTCAAAAACCTAAGGTTAAGTCGCCCTCGCTCGAGCGAGGGCGACTTAACCTTAGG were annealed and cloned into the plasmid “pLenti CMV Hygro DEST (w117-1)” (Addgene 17454). Lentiviral particles were produced according to Campeau et al. (8). Caco2BBe cells were infected with lentiviral particles supplemented with 4 µg/mL protamine sulfate, then selected with 150 µg/mL hygromycin B. Cultures were analyzed after 2 wk of selection.

For functional analyses, RT-qPCR and Western blotting, cells were seeded on Transwell polyester membrane of pore sizes 0.4 µm or 3 µm (Corning, Kaiserslautern, Germany) and grown 7–10 days to postconfluence. The transepithelial electrical resistance (TEER) of the monolayers in inserts was measured during the culture period by EVOM2 volt-ohm meter (World Precision Instruments) and calculated as Ω/cm^2^ of the growth area.

### Proliferation and Adhesion Assays

Cell proliferation in subconfluent cultures was quantified by BrdU cell proliferation assay kit (Merck), per manufacturers’ protocol. For cell adhesion assay, cultures at 50%–70% confluency were detached by trypsin-EDTA solution (Sigma-Aldrich), washed, counted, and resuspended in complete culture medium. 25,000 cells per well were seeded in 100 µL medium per well in 96-well flat-bottom plates. Cells were incubated for 24 h in cell culture incubator, then unattached cells were gently removed by two PBS washes. Adherent cells were fixed in 4% paraformaldehyde in PBS for 20 min, washed again, and blocked in 0.1% bovine serum albumin solution in PBS for 5 min at room temperature. Samples were stained with 50 µL of 0.1% crystal violet solution for 15 min, excess dye was removed by multiple washes with deionized H_2_O, and excess water was blotted by inverting the plate on absorptive paper for several minutes. Finally, the contents of the wells were solubilized with 100 µL of 1% SDS in H_2_O for 30 min at room temperature with gentle shaking, and the optical density of the samples was measured at 590 nm in a BioTek Epoch microplate reader (Agilent). Readouts were normalized to the average of the wild type for illustrations and analysis. Negative controls included wells containing only medium without cells, which were treated similarly and used as blanks. Proliferation rate between culture *days 2* and *14* was determined by crystal violet assay as used in the cell adhesion assay. To prepare 0.1% crystal violet solution, 1% crystal violet (Carl Roth) solution was prepared in methanol, then diluted 10-fold with deionized H_2_O and filtered to remove precipitants.

### RT-qPCR Analysis

Total RNA was extracted with RNeasy Mini Kit (Qiagen) and reverse transcribed to cDNA with RevertAid First Strand cDNA Synthesis Kit (Thermo Fisher Scientific) and oligo dT primers. Quantitative PCR was performed with a Rotor-Gene Q system (Qiagen) using qPCRBIO SyGreen Mix Lo-ROX (Nippon Genetics, Dueren, Germany), 10 ng of total cDNA, and 500 nM each of forward and reverse primers (Table 1).

**Table 1.** Name, brief description, and sequence of the primers for RT-qPCR of the genes that were analyzed in this study

Gene	Product; Description	Primer sequence for RT-qPCR
*SLC26A2*	Solute carrier family 26 member 2; apical sulfate/oxalate/Cl^−^ exchanger of intestinal mucosa	5´- CAGCCACATTAGCCTCTCATT 5´- CTGGTACTTTGGGTGGCATAA
*SLC26A3*	Solute carrier family 26 member 3; apical Cl^−^/HCO_3_^−^ exchanger of intestinal mucosa	5´- CCAGCGTCTATTCCCTCAAAT 5´- TCCCAGCAAATCCTCTGAATAC
*SLC26A6*	Solute carrier family 26 member 6; apical Cl^−^/HCO_3_^−^ exchanger of intestinal mucosa	5´- AGAAACTGCTCAAGAAGCAGGA 5´- CCATCTTATCTCCTGAGCTCACC
*SLC4A2 (AE2)*	Solute carrier family 4 member 2; basolateral Cl^−^/HCO_3_^−^ exchanger of intestinal mucosa	5´- GGGTGTCGGAGCTGATTATG 5´- CTGCTACAGAACGAGAAGAAGG
*SLC9A2 (NHE2)*	Solute carrier family 9 member 2; apical Na^+^/H^+^ exchanger of intestinal mucosa	5´- TGTCTACCGTGGGCAAGAAC 5´- AACGCAAAACAGATGGCACC
*SLC9A3 (NHE3)*	Solute carrier family 9 member 3; apical Na^+^/H^+^ exchanger of intestinal mucosa	5´- ACCGTGCGCTACACCATGAAGATG 5´- ATGCGGTAGCGGTTCAGAAGCC
*SLC9A8 (NHE8)*	Solute carrier family 9 member 8; apical/organellar Na^+^/H^+^ exchanger of intestinal mucosa	5´- TGGAGTTTGGCATGATGATCAT 5´- GTCTGCTGCATGAGGATCTG
*RPLP0*	Ribosomal protein lateral stalk subunit P0; Reference gene	5´- ACGGATTACACCTTCCCACT 5´- CGACTCTTCCTTGGCTTCAAC

### Immunofluorescence and Confocal Microscopy

Differentiated cultures grown on Transwell inserts were fixed with 2% paraformaldehyde solution in PBS for 30 min at room temperature, washed with PBS, and then blocked and permeabilized in PBS containing 5% normal goat serum (Sigma-Aldrich) and 0.2% Triton X-100 for 30 min. The concentration of Triton X-100 in blocking solution was reduced to 0.1% to dilute primary and secondary antibodies. Primary antibodies against SLC26A3 (1:100, Cat. No. sc-376187, Santa Cruz Biotechnology), NHE3 (1:500, Cat. No. NBP1-82574, Novus Biologicals), ZO-1 (1:200, Cat. No. 61-7300, Invitrogen), E-cadherin (1:200, Cat. No. 3195, Cell Signaling Technology), or Occludin (1:200, Cat. No. 33-1500, Thermo Fisher Scientific) were incubated with samples overnight at 4°C. After washing with PBS containing 0.1% Triton X-100, samples were exposed 1 h at room temperature to a mixture of secondary antibodies (goat anti mouse 488, Cat. No. A11029, and goat anti-rabbit 568, Cat. No. A11011, Invitrogen), DAPI to stain nuclei and Phalloidin-iFluor 647 Reagent to stain F-actin. After washing, samples were mounted using Immunoselect antifading mounting medium (DIANOVA # SCR-038447) and one layer of double-sided adhesive tape (Cat. No. 05338, Tesa, Norderstedt, Germany) was used as spacer between the slide and the coverslip. Confocal images were obtained by a TCS SP8 microscope (LEICA Microsystems, Mannheim, Germany) with a 63× oil immersion objective and analyzed by LAS X (LEICA Microsystems) and Fiji software.

### Transmission Electron Microscopy

Differentiated monolayers grown on Transwell inserts were fixed with 1.5% formaldehyde and 1.5% glutaraldehyde in 150 mM HEPES, pH 7.35 for 30 min. Processing, sectioning and mounting of samples were carried out at the core facility for electron microscopy at Hannover Medical School according to Dawodu et al. (14). Transmission electron microscopy images were obtained by a Morgagni TEM (FEI) equipped with a side mounted Veleta CCD camera and analyzed by Fiji software (15). Nine sections prepared from three independent cultures where analyzed for each of SLC26A3 WT or KO cultures.

### Western Blotting

Western analysis was performed only with antibodies for which immunospecificity was confirmed by knockout, knockdown, or transgenic overexpression in a Caco2BBe cell line (see Supplemental Fig. S1 for AE2 specificity confirmation). Differentiated cultures grown in culture flasks or on Transwell inserts were used for Western blotting as previously described (40). Primary antibodies against AE2 [rabbit polyclonal anti-SA6 peptide, 1:2,000 (16)], SLC26A3 (1:500, Cat. No. sc-376187, Santa Cruz Biotechnology), NHE3 (1:1,000, Cat. No. NBP1-82574, Novus Biologicals), claudin-2 (1:1,000, Cat. No. 32–5600, Invitrogen), E-cadherin (1:200, Cat. No. 3195, Cell Signaling Technology), ZO-1 (1:200, Cat. No. 61-7300, Invitrogen), or β-actin (1:2,000, Cat. No. 3700, Cell Signaling Technology) and HRP-conjugated secondary antibodies (1:10,000, Cat. No. G-21040 or G-21234, Invitrogen) were used to detect the bands. Samples were developed with ECL solution (Cat. No. RPN2209, Cytiva) and imaged and analyzed with a Fusion FX device equipped with Fusion-Capt Advance software (Vilber Lourmat, Eberhardzell, Germany).

### Fluorometry

Intracellular pH (pH_i_) at steady-state conditions and rates of Cl^−^/HCO_3_^−^ exchange were determined in differentiated Caco2BBe cultures by ratiometric fluorimetry using the intracellular pH-sensitive dye BCECF-AM (17). Solutions were prewarmed and gassed with carbogen (solutions I, II, and III; Table 2) in overhead reservoirs placed 30 cm higher than the microscope stage and selectively perfused into the cell chamber using a computer-controlled gravity-flow perfusion control system (ALA Scientific Instruments) at 1 mL·min^−1^ flow rate. Except for the segment beyond the flow-control valve, all tubing was gas-impermeable. A two-channel inline heater system (Warner Instruments) maintained perfusate temperature before entry into the chamber. Since the perfusion system introduced a minor loss of PCO_2_ [from 40 mmHg in the reservoir to ∼36.5 mmHg at the chamber input as measured by ABL5 blood-gas analyzer (Radiometer Medical) and calculated separately by Henderson–Hasselbalch equation], buffer bicarbonate concentration was set to 22 mM to achieve buffer pH of 7.4 in the assay chamber.

**Table 2.** Composition of solutions used for fluorometry

	Buffer I with Cl	Buffer II without Cl	Steady-State pH_i_	Calibration Buffers
CaCl_2_			1.5	1.2
Calcium gluconate	1.2	1.2		
Glucose	10	10	10	10
HEPES	10	18.5	10	10
K_2_HPO_4_	2.4	2.4	2.4	2.4
KCl			1.5	20
Potassium gluconate				110
KH_2_PO_4_	0.4	0.4	0.4	0.4
Mannitol	12.6			
Magnesium gluconate	1.2	1.2		
MgSO_4_			1	1.2
NaCl	110		116	20
Sodium gluconate		110		
NaHCO_3_	22	22	22	

Concentrations are in mmol·L^−1^. The pH of the calibration buffer was adjusted to the desired value by addition of HCl or NaOH.

Cl^−^/HCO_3_^−^ exchange rates were measured in differentiated cultures grown on Transwell polyester membrane inserts of 3 µm pore size. The filter was carefully sliced from the insert and mounted in a custom-made double perfusion chamber that provided separate apical and basolateral perfusion compartments. The chamber was placed on the stage with cells facing down, perfused apically with solution I to maintain cells, and loaded with 10 µM BCECF-AM dye in solution I for 30 min at room temperature from the basolateral side. After loading, the basolateral side of the chamber was also sealed and perfused with solution I. Fluorescent images were collected with a fluorescent microscope (Observer.A1, Zeiss) equipped with a long-distance ×20 objective, a VisiChrome high-speed polychromator system (Visitron systems), and a CCD digital camera (CoolSNAP HQ). Samples were sequentially excited at 445 nm (pH-insensitive) and 495 nm (pH-sensitive) for 100 ms each, and emissions at 530 nm (25 nm bandwidth) were collected and analyzed. After initial equilibration of samples by perfusing both apical and basolateral chambers with solution I to achieve a stable baseline, both sides were perfused with Cl^−^-free solution II to alkalinize the cells. Thereafter, sequential substitution of solution II with solution I was carried out in either apical-basolateral or basolateral-apical order, to evaluate the Cl^−^/HCO_3_^−^ exchange rate at each side of the cells in the absence (first recovery) or presence (second recovery) of Cl^−^ at the opposite side. Finally, intracellular pH was clamped to calibration buffers (Table 2) using the high K^+^/nigericin method (17, 18) and intracellular pH was calculated from the ratio of pH-sensitive/pH-insensitive emission after subtracting background fluorescence from the raw data. Cl^−^/HCO_3_^−^ exchange rate for each side was determined as the steepest ΔpHi/Δt after Cl^−^-recovery.

Steady-state pH_i_ of the samples was determined by perfusing apical and basolateral sides with solution III as explained above to obtain a stable base line for at least 20 min and then calibrated accordingly.

### Surface Fluid pH Measurement

Surface pH of Caco2BBe cultures was determined using a semi-automated fluorescent plate reader assay introduced by Saint-Criq et al. (19). This method was developed to measure the pH of airway epithelial cell monolayers at the apical surface, using a combination of pH-insensitive AlexaFluor488-dextran and pH-sensitive pHrodo-dextran fluorescent dyes.

We seeded and maintained WT and SLC26A3-KO clones of Caco2BBe cells on Transwell inserts for 24-well plates and used 9- to 10-day postconfluent cultures for experiments. On the day of experiment, the culture media was replaced with fresh complete medium. Couple of hours later, cultures were washed with sterile filtered HCO_3_^−^ containing Krebs buffer solution (HCO_3_^−^_KRB, composition in mM, 25 NaHCO_3_, 115 NaCl, 5 KCl, 1 CaCl_2_, 1 MgCl_2_ and 5 d-glucose) and transferred to a new 24 well plate containing 400 µl HCO_3_^−^_KRB in each well. The background intensity for individual inserts was measured in a Spark fluorescent plate reader (Tecan, Crailsheim, Germany). The device was prewarmed at 37°C with 5% CO_2_. Excitation filters for 485 nm and 535 nm wavelengths and emission filters for 535 nm and 590 nm wavelengths were used to measure pH-insensitive and pH-sensitive fluorescent intensities respectively. The optimal Z-position and gain values were already determined and kept constant for all experiments. After the background intensities were measured, 2 µg of AlexaFluor488-dextran and pHrodo-dextran dyes (Thermo Scientific, Darmstadt, Germany) equilibrated in 10 µL of glucose-free HCO_3_^−^_KRB was added to the apical surface of the cells, and the plate was returned to the plate reader for incubation and real-time measurements with 10-min intervals. Where indicated, this composition was also supplemented with a combination of 1 µM tenapanor (Adooq Bioscience, Irvine, CA) to inhibit NHE3 activity and 3 µM HOE642 (kindly provided by Sanofi-Aventis; Frankfurt, Germany) to inhibit NHE8 activity (20), or with 1 µM tenapanor only. At the end of the experiment, two steps of in situ pH calibrations at pH 8 and pH 6 were performed, each for 1 h in the absence of CO_2_ injection. For this purpose, highly buffered calibration solution containing 86 mM NaCl, 5 mM KCl, 1.2 mM CaCl_2,_ and 1.2 mM MgCl_2_, plus 100 mM Tris for pH 8 or 100 mM MES for pH 6 were used. For the first calibration, HCO_3_^−^_KRB was aspirated from the lower chamber, and then 50 µL and 400 µL of calibration buffer pH 8 were added to the upper and lower chambers respectively. For the next calibration, the upper and the lower solutions were aspirated completely and the chambers were washed with pH 6 calibration buffer. Then 60 µL pH 6 calibration buffer containing 2 µg of each of the fluorescent dyes were added to the upper chamber and 400 µL pH 6 calibration buffer was added to the lower chamber, and measurement was resumed. For data analysis, mean background was subtracted from mean value for each data point at each wavelength, and the ratio between pH-sensitive and pH-insensitive fluorescence was calculated. Fluorescent ratios were then converted to pH values using individual calibration curves for each sample.

### Electrophysiological Measurements of Ion Transport

Differentiated Caco2BBe-WT and KO cultures grown to confluency on 0.33 cm^2^ Transwell inserts of 0.4 µM pore size (Corning, Kaiserslautern, Germany) were used for Ussing chamber assays. The inserts were cut and mounted in the easy-mount Ussing chamber (easy-mount system, Physiologic Instruments) using the P2302T slider (Physiologic Instruments, San Diego, CA) with KCl-agar-filled fine tip electrodes (Physiologic Instruments, P2020-S Electrode Set). The samples were continuously perfused with luminal buffer (in mM: 106.5 NaCl, 20 NaHCO_3_, 2.25 KCl, 1.2 MgSO_4_, 2 CaCl_2_, 2.25 KH_2_PO_4_, 10 mannitol, and 10 Na-gluconate) and serosal buffer (in mM: 106.5 NaCl, 20 NaHCO_3_, 2.25 KCl, 1.2 MgSO_4_, 2 CaCl_2_, 2.25 KH_2_PO_4_, 10 glucose, and 10 Na-pyruvate), both gassed with carbogen (95% O_2_/5% CO_2_), and maintained at 37°C to provide a pH of 7.4. After obtaining baseline parameters for 40 min, drugs including amiloride (10 µM, luminal), forskolin (10 µM, serosal) plus IBMX (100 µM, serosal), UTP (100 µM, luminal), and bumetanide (100 µM, serosal) were added to the bath as indicated. Transepithelial resistance (Rte) and transepithelial voltage (Vte) were recorded every 6 s using a custom-made voltage/current clamp apparatus (Klaus Mussler, Aachen, Germany). Short-circuit current (I_sc_) was determined according to Ohm’s law (where I_sc_ is equal to Vte divided by Rte). To calculate ΔI_sc_, the average response peak was subtracted from the baseline average measured 1–2 min before drug addition.

### Data Analysis and Statistics

Microsoft Excel 2016 and GraphPad Prism version 8.0.2 (GraphPad Software Inc., San Diego, CA) software were used for data analysis and visualization. Data are presented as means ± SE. Statistical significance, as determined by *P* values from an unpaired, two-tailed parametric Student’s *t* test, or from ANOVA, is indicated as ns (not significant) *P* > 0.05, *P ≤ 0.05, **P ≤ 0.01, or ***P ≤ 0.001.

## RESULTS

### Generation and Characterization of SLC26A3-Deficient Caco2BBe Cells

**Figure 1.**
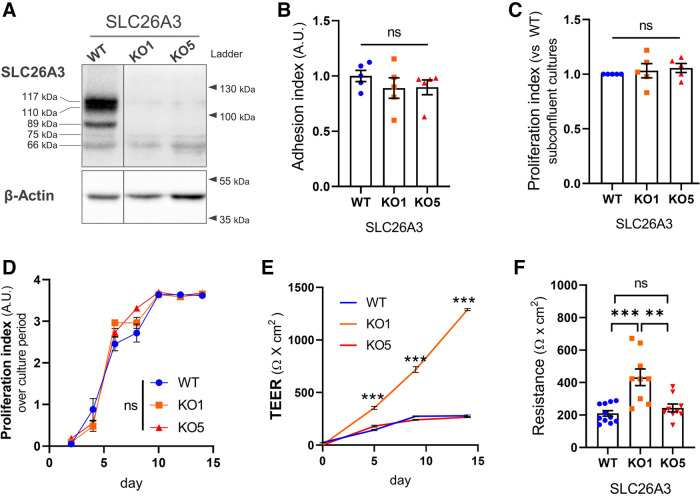
Characterization of SLC26A3 wild-type and knockout Caco2BBe cells. *A*: protein lysates from differentiated SLC26A3 wild-type (WT) and knockout (KO#1 and KO#5 clones) Caco2BBe cells were analyzed by Western blotting for protein expression of SLC26A3 using a commercially available antibody (see materials and methods). β-Actin signal was used as a loading control. The molecular masses (at *right*) are those of the protein ladder; those at *left* are calculated. *B*: cell adhesion index of SLC26A3-WT and KO Caco2BBe cells at 24 h postseeding (crystal violet assay). Mean optical densities were normalized to the mean of SLC26A3-WT. *C*: proliferation rates of subconfluent cultures were measured by BrdU assay and normalized to the values for SLC26A3-WT cells. *D*: proliferation indices of different samples throughout the culture period were determined by crystal violet assay, and mean optical densities are expressed in arbitrary units (AU). *E* and *F*: transepithelial electrical resistance (TEER) of the Caco2BBe cultures on Transwell inserts was measured throughout the culture period by EVOM volt-ohm meter and chopstick electrodes (*E*) or at the end of the culture period by Ussing chamber device (*F*) and are expressed as Ω/cm^2^ growth area. Values for SLC26A3-KO#5 and WT cells are comparable but are different from those of the KO#1 cells. SLC26A3-KO#1 and KO#5 cells are single-cell clonal isolates of wild type Caco2-BBe. *n* ≥ 5; ANOVA, ns *P* > 0.05, ** *P* ≤ 0.01, *** *P* ≤ 0.001.

Expression of SLC26A3 in Caco-2 cells was prevented by CRISPR-Cas9 targeting of *SLC26A3* exon 3, using an all-in-one CRISPR-Cas9 construct encoding EGFP in tandem with Cas9. Two days posttransfection, single GFP-positive Caco-2 cells were sorted and plated to form spatially separated clonal colonies. Among five selected clones, SLC26A3-knock out (KO) was confirmed by Sanger sequencing and Western blotting in clones KO1 and KO5 (Fig. 1 and Supplemental Fig. S1). Anti-SLC26A3 antibody detected major immunospecific SLC26A3 signals at 89 kDa (immature form) and at 110 and 117 kDa (mature forms) present in wild-type (WT) and absent in KO clones. In contrast, several fainter, nonspecific signals (115, 75, and 66 kDa) were present in both WT, SLC26A3-KO#1, and KO#5 clones (Fig. 1*A*).

Chromosomal instability in colon carcinoma-derived Caco2 cells has likely promoted genetic and epigenetic drift during multiple passages and resulted in subclonal populations that differ in physiological or morphological characteristics from each other (21). The Caco2BBe subclone of the Caco2 cells is therefore considered to be “pseudoclonal.” Single-cell isolates such as the SLC26A3-KO#1 and KO#5 clonal lines may, therefore, differ from the “pseudoclonal” Caco2BBe cells. Moreover, off-target effects of the CRISPR-Cas9 approach may additionally influence cell physiology. Therefore, SLC26A3-KO#1 and KO#5 clones were compared with the maternal cell line by several criteria. To test cell-matrix adhesion capability of SLC26A3-KO and WT clones, cells from subconfluent cultures were detached with trypsin-EDTA, resuspended in complete medium, and cultured for 24 h in a 96-well plate. After washing off nonadherent cells, the proportions of adherent cells were quantified by crystal violet stain. The cell adhesion indices of WT cells and the two KO clones were indistinguishable (Fig. 1*B*).

The BrdU proliferation indices of both KO clones were comparable to that of wild-type cells (Fig. 1*C*), and remained so during increasing times in culture (Fig. 1*D*). However, transepithelial resistance (TEER) in KO1 monolayers increased faster and to higher levels than in monolayers of KO#5 or WT cells (Fig. 1*E*). As the SLC26A3-KO#5 Caco2BBe clone exhibited TEER and morphology indistinguishable from those of WT cells, we selected the KO#5 clone for further investigation.

### Morphological Features of the SLC26A3-KO Caco2BBe Cells in Comparison to the WT Cells

**Figure 2.**
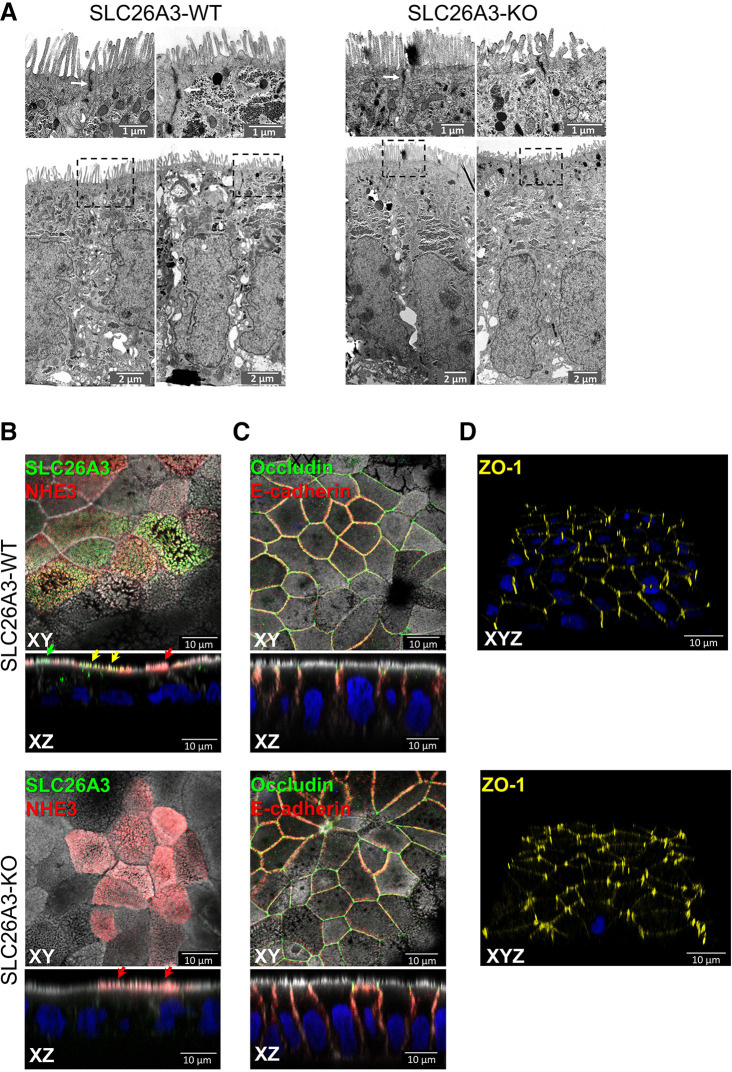
Morphological features of SLC26A3-KO Caco2BBe cells and WT cells. *A*: electron micrographs of SLC26A3-KO clones showed WT intestinal epithelial cell phenotype including apicobasal polarization, cell shape, shape and length of microvilli, and appearance and position of tight-junctions. In the upper panels showing enlarged views of the regions within the dashed squares, the tight junctions are marked by white arrows. *B* and *C*: *XY* and *XZ* confocal sections of SLC26A3-WT and KO cells showing immunofluorescence localization of SLC26A3 and NHE3 ion transporters, tight junction marker occludin and adherens junction marker E-cadherin. The phalloidin signal for F-actin is shown in gray, and nuclei are stained with DAPI (blue). In *XZ* sections, green, red, and yellow arrows, respectively, indicate apical surface localizations of SLC26A3, NHE3, and colocalization of SLC26A3 plus NHE3. *D*: three-dimensional reconstructions of ZO-1 signals in SLC26A3-WT and KO monolayers show comparable ZO-1 localizations.

Electron micrographs of SLC26A3-KO#5 monolayers showed WT IEC phenotypes, including apicobasal polarization, shape and length of microvilli and appearance and position of tight-junctions (Fig. 2*A*). Confocal laser immunolocalization studies of WT and KO monolayers revealed similar expression and localization of the IEC marker proteins villin, E-cadherin and NHE3 (Fig. 2, *B* and *C*). Microvillar and cellular expression patterns of both SLC26A3 and of NHE3 exhibited some heterogeneity among individual WT cells. The cells were studied at 9–10 days post confluency, prior to terminal differentiation and maximal NHE3 expression. This growth condition is suboptimal for fluorimetric measurement of cell pH_i_, perhaps reflecting cellular heterogeneity of dye loading, as well as matrix accumulation on and propensity for detachment from the filter substrate.

In both WT and KO monolayers tight junctional marker protein ZO-1 was more abundant and extended further towards the basolateral filter substrate at multicellular junctions than at bicellular interfaces (Fig. 2*D*). This observation contrasts with previously reported perturbations of ZO-1 localization in SLC26A3-KO cells (22), which may represent differences in XZ planes through the monolayers selected for presentation. The above electron micrographs and immunofluorescence images collectively confirm similar morphological phenotypes of WT and SLC26A3-KO#5 Caco2BBe cell monolayers.

### Electrophysiological Comparison of WT or SLC26A3-KO Caco2BBe Monolayers

**Figure 3.**
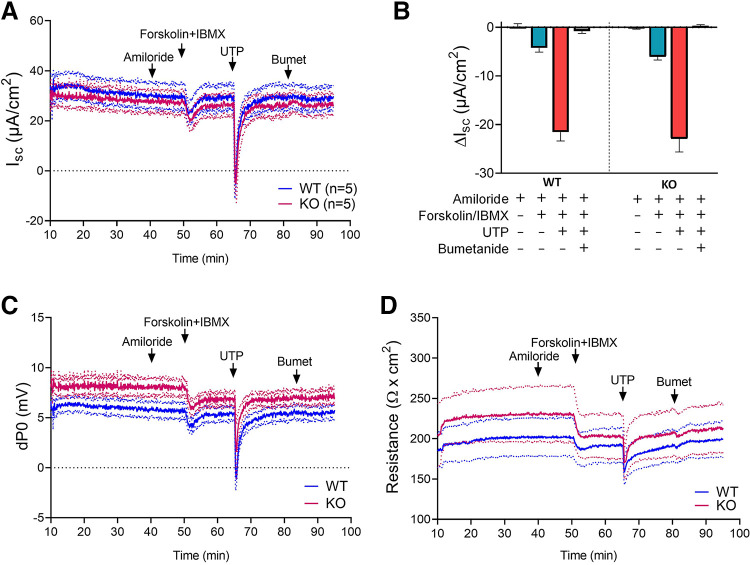
Electrophysiological comparison of differentiated SLC26A3-WT and KO Caco2BBe monolayers in Ussing chamber. *A* and *B*: comparable short-circuit current responses (Isc) of SLC26A3-WT and KO monolayers to additions of amiloride (10 µM luminal), forskolin (10 µM, serosal) plus IBMX (100 µM, serosal), UTP (100 µM luminal), or bumetanide (100 µM serosal); corresponding traces of transepithelial potential difference and electrical resistance are shown in *C* and *D*, respectively. Dotted traces indicate upper and lower limits of SEM values. *n* ≥ 5, ANOVA. There was no statistically significant difference in the corresponding responses between WT and KO.

To compare the electrophysiological properties of SLC26A3-WT and KO clones, monolayers were studied in classic Ussing-chamber systems at 9–10 days postconfluency. As shown in Fig. 3, *A*–*D*, SLC26A3-KO#5 and WT cells I_sc_ exhibited similar responses to adenylyl cyclase activator forskolin (FSK) and to purinergic receptor agonist UTP. The small FSK-induced ΔI_sc_ resembled that of differentiated colonic organoid-derived monolayers, consistent with progressive downregulation of the apical CFTR anion channel and the basolateral NKCC1 sodium/potassium/chloride cotransporter during monolayer differentiation (12, 23). The larger ΔI_sc_ induced by UTP than by FSK differed from that in terminally differentiated monolayers derived from colonic organoids or in mouse intact colonic mucosa (23).

### Greatly Reduced Surface pH in SLC26A3-KO Clone Compared to WT Cells

**Figure 4.**
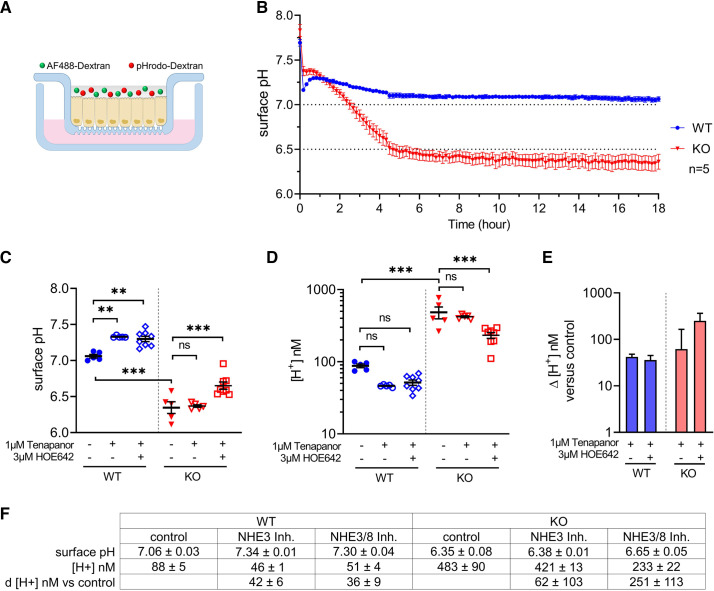
Lack of SLC26A3 in differentiated Caco2BBe monolayers strongly affects apical surface acid/base balance. *A*: schematic of the ratiometric technique to measure apical compartment pH of epithelial monolayers using pH-sensitive (pHrodo red) and pH-insensitive (AlexaFluor 488) dyes coupled to dextran. *B*: time course of change in apical surface pH for differentiated Caco2BBe monolayers shows much lower steady-state values for SLC26A3-KO cells than for WT. *C*: inhibition of NHE3 with 1 µM tenapanor and of NHE8 with 3 µM HOE642 increases monolayer apical surface pH for both SLC26A3-WT and KO, while maintaining significant differences between WT and KO values. *D*: Apical surface proton concentrations calculated for each condition based on pH values in *C*, by equation [H^+^] = 10^−pH^. *E*: NHE3/8 inhibition-dependent difference in apical surface proton concentrations for SLC26A3-WT and KO monolayers. *F*: summary of data from *C*–*E*. *n* ≥ 5, Student’s *t* test, **P ≤ 0.01, ***P ≤ 0.001.

A dual fluorophore technique to measure apical surface pH (the extracellular pH immediately adjacent to the apical cell membrane) introduced for airway cell monolayers in air-liquid culture (19) was adapted to Caco2BBe monolayers (Fig. 4*A*). The apical surface pH recorded over 18 h (Fig. 4*B*) in WT cells was initially high, reflecting the 25 mM HCO_3_^−^ in the pH 7.4 medium, but equilibrated and stabilized in a 5% CO_2_^−^containing atmosphere at ∼7.1. In contrast, apical surface pH in SLC26A3-KO monolayers fell continuously over several hours to a plateau value of ∼6.4 (Fig. 4, *B* and *C*). The large difference in apical surface pH between WT and KO monolayers was stable (Fig. 4*C*). The results demonstrate that SLC26A3 function is essential to maintain WT apical surface pH.

**Figure 5.**
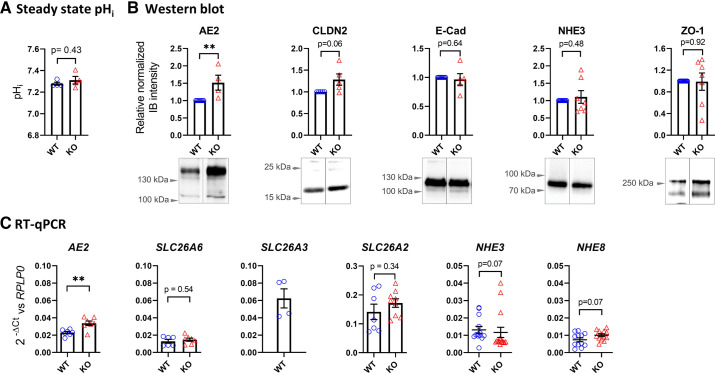
*A*: steady-state pHi was determined fluorometrically during constant bilateral perfusion with CO_2_/HCO_3_^−^-containing perfusion buffers of pH 7.4. No significant difference was observed in the steady-state pH_i_ of WT and SLC26A3-KO cells. *B*: immunoblots of cell lysates from WT and SLC26A3-KO monolayers detecting protein abundance of AE2, Claudin 2 (CLDN2), E-cadherin (E-Cad), NHE3 and ZO-1. Immunoblot band intensities in SLC26A3-KO lysates were normalized to their paired signals in WT lysates. Resulting values were normalized to their corresponding beta-actin loading controls (not shown). AE2 protein expression was significantly increased in SLC26A3-KO vs. WT monolayers. *C*: RT-qPCR abundance of *AE2*, *SLC26A6*, *SLC26A2, NHE3*, and *NHE8* mRNAs in SLC26A3-WT and KO monolayers and of *SLC26A3* mRNA in WT cells. As for protein expression, only *AE2* mRNA expression was significantly elevated in SLC26A3-KO vs. WT monolayers. *n* ≥ 4, Student’s *t* test, **P ≤ 0.01.

Differentiated Caco2BBe WT and KO cells expressed relatively high levels of sodium/hydrogen exchangers NHE3 and NHE8, among the major apical proton extruders of the apical surface of the differentiated intestinal epithelium (Fig. 5). To assess apical NHE regulation of surface pH in the presence or absence of SLC26A3, we included in the apical fluid 1 µM of the NHE3-specific inhibitor tenapanor, or 1 µM tenapanor plus 3 µM HOE642 to also inhibit NHE8. The addition of tenapanor to WT cells increased surface pH, consistent with a reduced luminal proton secretion by the inhibited NHE3 (Fig. 4, *D*–*F*). Additional inhibition of NHE8 by HOE642 did not further increase surface pH. In contrast, addition of apical tenapanor to SLC26A3-KO cells failed to change apical surface cell pH, but the supplemental addition of HOE642 (surprisingly) increased surface pH. The reduction in apical surface proton concentration by combined inhibition of NHE3 and NHE8 was greater in SLC26A3-KO than in WT monolayers (Fig. 4, *D*–*F*), likely reflecting stimulation of KO cell apical NHEs by the higher subapical acid load produced by the lower surface pH of KO monolayers.

### Steady-State pH_i_ Does Not Differ between WT and SLC26A3-KO Cells

Steady-state pH_i_ was determined fluorometrically during constant bilateral perfusion with CO_2_/HCO_3_^−^ containing perfusion buffers of pH 7.4 (Fig. 5*A*). No significant difference was noted in steady-state pH_i_ of differentiated WT and SLC26A3-KO monolayers, despite the dramatic difference in their maximal rates of apical HCO_3_^−^ export (Fig. 4). This was unexpected, as colonic crypts from *slc26a3*^−/−^ mice and WT littermates exhibited significant difference in steady-state pH_i_ when isolated from the crypt mouth but not from the crypt base (7). We therefore tested possible explanations for this finding.

### Expression of Other Acid/Base Exchangers in WT and SLC26A3-KO Cells

Expression of several membrane and junctional proteins was investigated by Western analysis of lysates of WT and SLC26A3-KO monolayers. Significant upregulation was found for the basolateral Cl^−^/HCO_3_^−^ exchanger AE2 that, like the apical SLC26A3, is an acid-loader (Fig. 5*B*). Immunospecificity of the AE2 signal in Western blotting was verified by comparing immune signals of *AE2* knockdown Caco2BBe to control monolayers (Supplemental Fig. S3). AE2 mRNA was similarly upregulated in SLC26A3-KO monolayers (Fig. 5*C*). WT and SLC26A3-KO cells both showed robust expression of SLC26A2 mRNA whereas expression levels of SLC26A6 mRNA were substantially lower (Fig. 5*C*). Both SLC26A2 and SLC26A6 are expressed at the luminal surface of the intestinal epithelium. SLC26A6 mediates Cl^−^/HCO_3_^−^ exchange (24, 25) and may contribute to residual apical Cl^−^/HCO_3_^−^ exchange activity in Caco2BBe cells as described below. However, SLC26A2 does not mediate Cl^−^/HCO_3_^−^ exchange, but rather exchanges sulfate for Cl^−^, OH^−^, and/or Ox^2−^ ions (26, 27). NHE3 protein and mRNA expression in WT and KO cells did not differ, and the same was true of NHE8 mRNA expression.

### Apical and Basolateral Cl^−^/HCO_3_^−^ Exchange in WT and SLC26A3-KO Monolayers

**Figure 6.**
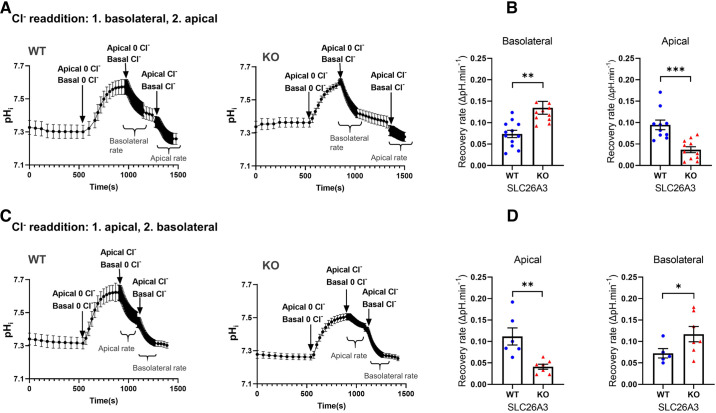
Rates of apical and basolateral Cl^−^/HCO_3_^−^ exchange in differentiated WT and SLC26A3-KO monolayers. After baseline steady-state pH_i_ was established, both apical and basolateral compartments were perfused with Cl^−^-free buffers. To differentially address apical and basolateral Cl^−^/HCO_3_^−^ exchange rates at maximal transepithelial chloride gradient, readdition of Cl^−^-containing buffer was performed in the basolateral-apical (pH_i_-traces in *A*, initial pH_i_ recovery rates in ΔpH_i_/min in *B*) or apical-basolateral (*C* and *D*) order. Rates were calculated from the steepest linear slopes of pH_i_-decline and extrapolated for 1 min. In the absence of SLC26A3, apical Cl^−^/HCO_3_^−^ exchange was reduced but not abolished, whereas basolateral Cl^−^/HCO_3_^−^ exchange rates (mediated at least in part by AE2) were enhanced (significantly in the basolateral-apical condition). *n* ≥ 5, Student’s *t* test, *P ≤ 0.05, **P ≤ 0.01, or ***P ≤ 0.001.

Apical and basolateral Cl^−^/HCO3^−^ exchange rates were determined in differentiated, high-resistance monolayers of WT and SLC26A3-KO Caco2BBe cells. Cl^−^ was simultaneously removed from both apical and basolateral baths, followed by sequential Cl^−^ readdition first to the apical bath and then to the basolateral (Fig. 6*A*), or vice versa (Fig. 6*C*) as shown in the representative pH_i_ traces. V_max_ was computed from the initial pH_i_ recovery rate immediately following Cl^−^ readdition to either basolateral (Fig. 6*B*) or luminal (Fig. 6*D*) perfusate following prior bilateral Cl^−^ removal (Fig. 6*B*). The pH_i_-recovery rates after Cl^−^-addition to the contralateral membrane (the second addition) were also calculated (*right panel* bar graphs in Fig. 6, *B* and *D*). These latter measured values may differ from the V_max_, as Cl^−^ has already re-entered the cell, reducing the driving force for incremental Cl^−^ entry. Experimental time intervals differed to achieve similar pH_i_ values before Cl^−^ readdition (pH_i_ was 7.58 ± 0.034 in WT and 7.57 ± 0.016 in KO cells at the time of first Cl^−^ readdition, but 7.39 ± 0.026 in WT and 7.36 ± 0.023 in KO cells at second readdition of Cl^−^). Initial pH_i_ recovery rates within the steepest linear pH_i_-decline were calculated and extrapolated to 1 min, as shown on the *y*-axes of Fig. 6, *B* and *D*.

The pH_i_ decline after basolateral Cl^−^ readdition likely represents AE2-mediated Cl^−^/HCO_3_^−^ exchange, whereas the pH_i_ decline after apical Cl^−^ readdition probably represents SLC26A3/A6-mediated Cl^−^/HCO_3_^−^ exchange. Basolateral Cl^−^/HCO_3_^−^ exchange rates are significantly higher in SLC26A3-KO than in WT monolayers. In contrast, apical Cl^−^/HCO_3_^−^ exchange rates are significantly lower in SLC26A3-KO than in WT monolayers, but not abrogated. Residual apical Cl^−^/HCO_3_^−^ exchange in SLC26A3-KO monolayers is likely mediated by SLC26A6 and, perhaps, by other base exchangers.

## DISCUSSION

The effects of chronic intracellular alkalosis in the absence of alterations of extracellular acid/base status have rarely been studied, as achievement of this state is experimentally demanding. Acute increases in pH_i_ rapidly activate compensatory ion transport mechanisms that extrude base from the cytoplasm, such as the Cl^−^/HCO_3_^−^ exchangers of the SLC4 and SLC26 gene families (28, 29). Chronic systemic alkalosis may accompany chronic hyperventilation (30) metabolic diseases (31) or genetic diseases such as congenital chloride diarrhea, in which intestinal enterocytes cannot efficiently extrude base (32), or cystic fibrosis, in which the kidney (among other tissues) cannot do so (33). Multiple adaptive mechanisms to systemic alkalosis have been explored (34–36), but chronic cellular responses to deletion of a major base extruder remain little investigated. The CRISPR/Cas9-mediated knockout (KO) of SLC26A3 in the self-differentiating intestinal cell line Caco2BBe, followed by the establishment of clonal KO cell lines, allows deletion of a specific protein without continuous antibiotic treatment, as required for short hairpin-mediated gene silencing. The high and inducible expression levels of multiple drug transporters have complicated or prevented efficient and persistent antibiotic-mediated downregulation of target genes (12, 20). Deletion of the SLC26A3 protein from our Caco2BBe cell clones was confirmed by Sanger sequencing, immunoblotting, and immunofluorescence data.

The reduction in apical Cl^−^/base exchange in SLC26A3-KO monolayers was substantial but incomplete. One potential candidate for the residual activity of KO monolayers is the Cl^−^/Ox^2−^/SO_4_^2−^/HCO_3_^−^ exchanger SLC26A6 (24, 25), whose mRNA is expressed in WT cells at lower levels than *SLC26A3*. However, the very low surface pH of the SLC26A3-KO cells argues against a robust HCO_3_^−^ output by SLC26A6 under the steady-state conditions of the surface pH measurements. We previously reported very acidic juxta-mucosal (apical surface) pH in the cecum and mid-distal colon of *slc26a3*^−/−^ mice in vivo (10). Although *SLC26A6* mRNA expression is higher in murine small intestine and cecum than in murine mid-distal colon, why the reduced mouse intestinal bicarbonate secretion of *slc26a3*^−/−^ mice is not accompanied by greater compensatory upregulation of SLC26A6-mediated bicarbonate secretion remains unexplained (10, 37).

Another potential candidate might be SLC26A2, whose mRNA is highly expressed in both Caco2BBe WT cells and SLC26A3-KO cells, but which has been considered a SO_4_^2−^/OH^−^/Cl^−^ exchanger or an SO_4_^2−^/Cl^−^/Ox^2−^ exchanger of the enterocyte luminal membrane (26, 27, 38). Our perfusion buffers for the determination of Cl^−^/HCO_3_^−^ exchange rates lacked added sulfate, but other anions transported by SLC26A2 may contribute to base exchange under conditions of a high chemical driving force for Cl^−^ exit.

Functional coupling of NHE3 and SLC26A3 has been described in Caco2BBe cells, with no apparent NHE-independent activity of either endogenous or transfected SLC26A3 (39). In our study, the cellular expression of SLC26A3 and NHE3 only partly overlaps, as previously noted in differentiated colonic organoids (40). Moreover, the heterogeneous NHE3 expression in our Caco2BBe WT cells [the product of clonal selection (41)] remained heterogeneous even in our clonal SLC26A3-KO cells derived from single-cell selection (Fig. 2 and Supplemental Fig. S2). However, our confocal images of the monolayer apical surface, which is of variable height, may not have captured the apical membrane of every imaged cell in a given *XY* plane or stack. Over the 3-wk period of postconfluency Caco2BBe cell differentiation, the apical expression of both NHE3 and SLC26A3 increases but remains heterogeneous. The apparently independent variation of SLC26A3 and NHE3 expression in Caco2BBe WT and SLC26A3-KO cells may represent yet undefined stochastic epigenetic control of transcription, translation, protein trafficking, and/or protein stability. Data suggesting at least some functional independence of NHE3 and SLC26A3 has emerged from jejunal perfusion studies carried out in anesthetized mice. Perfusion of *slc26a3*^−/−^ jejunum with unbuffered solution resulted in strong acidification of mucosal fluid, whereas unbuffered perfusion of *slc9a3*^−/−^ mice resulted in mucosal alkalinization. However, both *slc26a3*^−/−^ and *slc9a3*^−/−^ mice exhibited decreased jejunal fluid absorption as compared with WT mice (37).

To gain further insight into SLC26A3 activity and its coupling to apical NHE activity under steady-state conditions, we adapted for Caco2BBe cells a technique originally developed to measure airway surface liquid pH (not grown with an air-liquid interface, but covered with a thin apical liquid layer). Comparison of surface pH in WT and SLC26A3-KO monolayers, in the absence or presence of NHE3 inhibitor or combined inhibition of NHE3 and NHE8 yielded unexpected results. Whereas luminal addition of the specific NHE3 inhibitor tenapanor increased surface pH in WT cells, the lower surface pH of the KO cells did not significantly change. This difference may reflect strong inhibition of NHE3-mediated Na^+^_o_/H^+^_i_ exchange by the high apical surface proton concentration of KO cells (42). The high proton concentration may also interfere with tenapanor binding, although no published data address this possibility. The surprising finding that the additional inhibition of NHE8 with 3 µM Hoe642 significantly increased the low surface cell pH of SLC26A3-KO monolayers, but not the higher surface cell pH of WT cells, might reflect increased surface trafficking and activity of NHE8, as well as the lower proton affinity of NHE8’s extracellular Na^+^ transport site than that of NHE3. Acidic extracellular pH can stimulate ion channel and organellar transport to the plasma membrane (43, 44), as well as NHE8 activity (45).

Of course, the pH_i_ is a major determinant of both NHE and SLC26A3 activities (29, 42, 46). The first report of *slc26a3*^−/−^ mice described strong upregulation of colonic NHE3 and ENaC expression, likely reflecting the profound hyperaldosteronism proposed as a compensatory mechanism driving increased fluid absorption despite absent luminal Cl^−^/HCO_3_^−^ exchange (6). We confirmed increased NHE3 and ENaC expression in *slc26a3*^−/−^ colonic mucosa but, surprisingly, virtually no fluid absorption was detected upon saline perfusion of mid-distal colon in anesthetized *slc26a3*^−/−^ mice (7). We later realized that fluid absorption was partially rescued when perfusate pH was kept neutral by increasing the buffer capacity of the perfusate. The originally used unbuffered saline perfusate had adopted the low pH of the mucosal surface (47). A second reason for the lack of functional rescue by NHE3 upregulation was the high pH_i_ of *slc26a3*-deficient terminally differentiated enterocytes, in which NHE3 was predominantly localized to the terminal web region, whereas WT enterocytes expressed more NHE3 along the length of the microvilli (37). When isolated colonic crypts or small intestinal villi were subjected to acid load imposed by ammonium prepulse, the subsequent NHE3-mediated proton extrusion rate was higher in enterocytes from *slc26a3*^−/−^ mice than from WT mice, suggesting redistribution of NHE3 to the *slc26a3*^−/−/^ terminal web region by its previously elevated enterocyte pH_i_.

In contrast, steady-state pH_i_ in clonal SLC26A3-KO#5 cells was no higher than in WT cells (whereas KO#1 cells exhibited both higher pH_i_ and higher transepithelial resistance, consistent with the absence of a major acid extruder as well as decreased trans-monolayer ion transport). We selected the KO#5 clone for further study to investigate compensatory mechanisms that allow for a steady-state pH_i_ similar to the WT cells despite the absence of a major apical base extruder. The possibility of a reduced activity of apical NHEs (less proton extrusion in the absence of SLC26A3) was not supported by either apical surface pH measurements or immunoblotting or immunostaining. Another possible mechanism may be increased basolateral base extrusion via basolateral Cl^−^/HCO_3_^−^ exchanger AE2. Indeed, *AE2* mRNA and proteins were both upregulated in KO#5 monolayers (Fig. 6). Although neither *SLC26A6* nor *SLC26A2* mRNAs were increased in KO#5 cells, the residual apical anion exchange documents persistent activity of one or more apical base extruders at least under conditions of maximal Cl^−^ gradient if not in the physiological [Cl^−^] at which apical surface pH was measured (Fig. 4).

Therefore, we compared the AE2-mediated fluorimetric base extrusion rate in WT and SLC26A3-KO5 cells at near-maximal driving force, by assessing the initial pH_i_-recovery rate after selective readdition of Cl^−^ to the basolateral perfusate. The re-acidification rate was indeed significantly higher in SLC26A3-KO#5 cells than in WT Caco2BBe cells.

What may explain the difference between SLC26A3-KO#5 cells with AE2-mediated compensatory base efflux preventing an alkaline pH_i_ secondary to deletion of the major apical Cl^−^/HCO_3_^−^ exchanger, and terminally differentiated *slc26a3*^−/−^ enterocytes of small intestinal villi and the colonic surface in situ, which display elevated pH_i_ (37)? One reason may be that the >60-fold upregulation of SLC26A3 in 4-day-differentiated human colonoids versus highly proliferative colonoids is accompanied by comparable downregulation of AE2 across the brief 5- to 7-day lifetime of native enterocytes (40), such that AE2 cannot compensate for absent SLC26A3. Caco2BBe cells, by contrast, although originally tumor-derived, are lineage-determined and differentiate much more slowly into absorptive enterocyte-like cells, never reaching the high mRNA and protein expression levels characteristic of terminally differentiated colonic enterocytes. Thus, *SLC26A3* mRNA expression is detectable in Caco2BBe cells only by *day 3* after seeding subconfluent cultures, and increases ∼10-fold by 7–9 days postconfluency. This ∼3-wk growth and differentiation process enables and/or requires adaptive changes in gene and/or protein expression across the Caco2BBe lifespan in culture. In particular, AE2 protein abundance is unchanged at 1- and 3-wk postconfluency. Differentiated enterocyte marker protein expression starts > 1-wk postconfluency (48, 49). This suggests that the AE2 expression is more stable over the time of differentiation (and induced to lower levels) in cultured Caco2BBe cells than in human colonoids. The increased expression and function of AE2 in SLC26A3-KO cells most likely prevents alkalinization of pH_i_ secondary to loss of SLC26A3.

The stable expression of AE2 in Caco2BBe cells may relate to their postconfluency maintenance of both proliferative capacity and continued differentiation. Some typical “crypt” markers such as CFTR are downregulated over time in culture of Caco2BB3 cells, whereas typical “surface” markers such as Slc26a3 or intestinal alkaline phosphatase are upregulated (12). However, neither magnitude of change is nearly so dramatic as observed during the much shorter differentiation time in colonoids (23), underlining the origin of Caco2BBe cells from a malignant tumor, with retention of some characteristics of cancer cells different from those of intestinal epithelium. A possibly related property is the markedly stronger Isc response of differentiated Caco2BBe monolayers to the stable ATP analogue UTP than to forskolin. The UTP-stimulated, purinergic receptor-mediated increase in [Ca^2+^]_i_ may in turn activate the TMEM16A channel, which is upregulated in colorectal cancer and in cancer cell lines (50). However, this response pattern is atypical of isolated intestinal colonic epithelium as well as of undifferentiated and differentiated monolayers derived from human colonoids (23). The results emphasize that the Caco2BBe spontaneously differentiating intestinal cell line, although highly valuable to understand intestinal transporter function, is nevertheless a tumor cell line with its own biology, and neither recapitulate all features of either crypt- or surface cells from small or large intestinal enterocytes, while retaining features of cancer cells.

In summary, we compared functional properties of a Caco2BBe cell line with a CRISPR/Cas9-mediated SLC26A3 deletion with those of WT Caco2BB3 cells. SLC26A3-KO cells exhibited a dramatically lower surface pH, a >70% reduction of apical Cl^−^/HCO_3_^−^ exchange activity, and transcriptional and functional upregulation of the basolateral Cl^−^/HCO_3_^−^ exchanger AE2, while proliferative capacity, time to confluency, transepithelial resistance and cellular morphology were comparable to those of the maternal cell line. As Caco2BBe cells can self-differentiate into an epithelial monolayer with features resembling native colonic epithelium (while also retaining aspects of malignant cells), these SLC26A3 knockout cells may prove a useful and informative addition to the armamentarium of reagents with which to better understand the regulation of ion transport functions in colonic enterocytes and colorectal cancer cells.

## DATA AVAILABILITY

Data will be made available upon reasonable request.

## SUPPLEMENTAL DATA

10.6084/m9.figshare.24487669 Supplemental Fig. S1: https://doi.org/10.6084/m9.figshare.24487669.

10.6084/m9.figshare.24805617 Supplemental Fig. S2: https://doi.org/10.6084/m9.figshare.24805617.

10.6084/m9.figshare.24487675 Supplemental Fig. S3: https://doi.org/10.6084/m9.figshare.24487675.

## GRANTS

The work was funded in part by Deutsche Forschungsgemeinschaft Grant FOR5046 SE460/22-1.

## DISCLOSURES

No conflicts of interest, financial or otherwise, are declared by the authors.

## AUTHOR CONTRIBUTIONS

M.A. and U.E.S. conceived and designed research; M.A., M.J., A.S., and R.X. performed experiments; M.A., M.J., A.S., and U.E.S. analyzed data; M.A., M.J., A.S., S.L.A., and U.E.S. interpreted results of experiments; M.A., M.J., and A.S. prepared figures; M.A., M.J., A.S., and U.E.S. drafted manuscript; M.A., A.S., S.L.A., and U.E.S. edited and revised manuscript; M.A., M.J., A.S., R.X., S.L.A., and U.E.S. approved final version of manuscript.
